# Classical Genetics Meets Next-Generation Sequencing: Uncovering a Genome-Wide Recombination Map in *Drosophila melanogaster*

**DOI:** 10.1371/journal.pgen.1003024

**Published:** 2012-10-11

**Authors:** Nadia D. Singh

**Affiliations:** Department of Genetics, North Carolina State University, Raleigh, North Carolina, United States of America; Stanford University, United States of America

**Figure 1:**
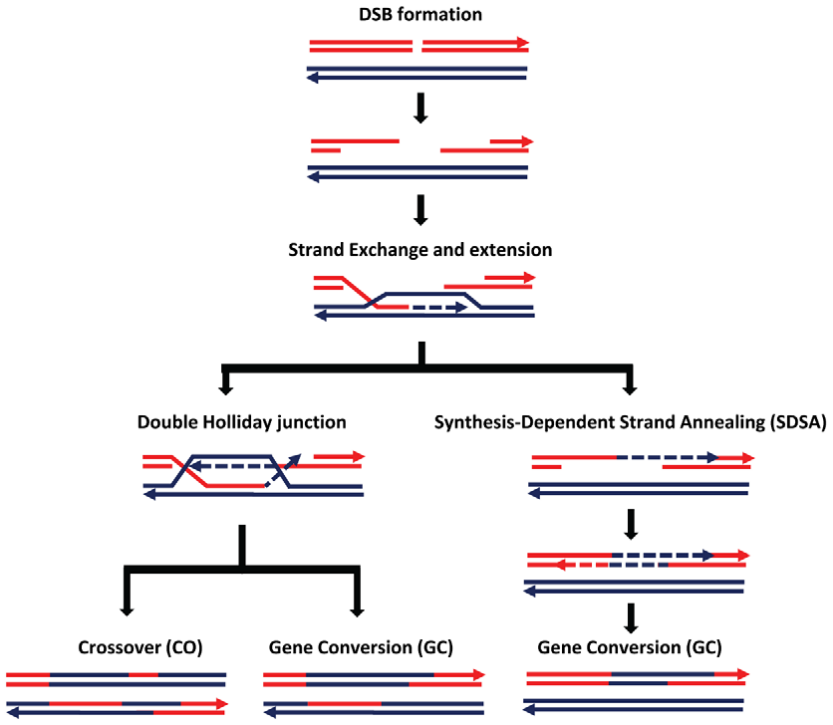
Schematic representation of the double-strand break (DSB) repair pathway and recombination from Comeron et al. [**15**]. Note that crossovers only result from resolution of a double Holliday junction, while noncrossover events (denoted here as “gene conversion” events) can result from both the SDSA pathway and resolution of a double Holliday junction.

Homologous recombination is a potent genetic force that impacts myriad aspects of genome evolution, from standing levels of nucleotide diversity to the efficacy of natural selection. Coarse-scale recombination rates have long been known to be variable, and much of the early work exploring this variation exploited *Drosophila melanogaster* as a model [1]–[5]. Yet, determining the scale and scope of intra- and inter-genomic variation in fine-scale recombination rate in Drosophila has proven quite challenging. Fine-scale recombination rate variation is well-described in humans, mice, and yeast, owing in part to techniques such as sperm typing and chromatin immunoprecipitation (for review, see [6]). However, the underlying biology of recombination in Drosophila (including the lack of crossing-over in males, a less punctate recombinational landscape, and the technical difficulties associated with isolating meiotically active cells from the female germline) has precluded the application of these techniques to Drosophila. Moreover, linkage disequilibrium–based approaches, which have enjoyed success in many systems (e.g., [7], [8]), have been hampered in Drosophila until recently by a lack of genome-wide polymorphism data. Though such data are increasingly available, the rapid decay of linkage disequilibrium in Drosophila (e.g., [9]) and possible rampant adaptation (e.g., [10]) may limit the accuracy and efficacy of such approaches. Consequently, previous work exploring fine-scale recombination rate variation in Drosophila has been limited to localized regions or one to two chromosomes (e.g., [11]–[14]). Not to be deterred, Comeron and colleagues couple the power of classical genetics with next-generation sequencing to provide for the first time a high-resolution recombination map of the *D. melanogaster* genome [15]. Both outcomes of the meiotic recombination process are captured therein: crossovers, which involve reciprocal exchange of genetic material, and noncrossovers, which result in non-reciprocal exchange (Figure 1).

To create this landmark map, Comeron and colleagues generated recombinant advanced intercross lines (RAIL), derived from eight crosses among twelve wild-derived lines. To accurately identify crossover and noncrossover events, haplotype rather than genotype data are required, and Comeron and colleagues use a clever technique to recover haplotypes. RAIL females were individually crossed to *D. simulans*, and the genomes of single hybrid progeny were sequenced with Illumina technology. Reads mapping to *D. simulans* were removed bioinformatically to reveal a haploid, meiotically produced *D. melanogaster* genome. In all, over 100,000 recombination events were localized with kilobase-level precision.

Certainly, this genome-wide recombination map will empower population genetic and molecular evolutionary studies in Drosophila for years to come. However, the sheer number of events catalogued combined with the resolution at which breakpoints could be mapped facilitates a great deal more than quantifying intra- and inter-genomic recombination rate variation. For instance, these data show that although crossover and noncrossover rates are both significantly variable genome-wide, rates of crossing-over are ten times more variable than noncrossover rates. In addition, crossing-over rates are variable among crosses, with the bulk of this variation being driven by regions of increased crossing-over revealed in some crosses but not in others. This is in contrast to previous work suggesting evolutionary conservation of fine-scale recombination rates in Drosophila [14]. Thus, the physical and temporal scales at which fine-scale recombination rates are conserved remain an open question. Another striking finding is that noncrossover and crossover rates are negatively correlated, and moreover, the noncrossover∶crossover ratio correlates negatively with nucleotide diversity. Indeed, the elegant simplicity of this experiment is in stark contrast to the rich complexity of the resulting data, with the results shedding unprecedented light on variation in the Drosophila recombinational landscape and providing new insights into the genetic and molecular bases of this variation.

These data should also allow us to address multiple aspects of the recombination process in an evolutionary context, building on recent advances in other systems. For example, the noncrossover∶crossover ratio has a considerable range, from 0.73∶1 in yeast [16] to 4∶1–15∶1 in humans [17], with *D. melanogaster* showing a ratio of ∼4∶1 [15]. What determines this ratio? Are different double-strand break resolution pathways (Figure 1) employed to different degrees in different systems, or has divergence in the proteins involved in these pathways generated this variation? Similarly, tract lengths associated with noncrossovers show marked variability, with a median length of 1.8 kb in yeast but much shorter tract lengths in humans (200–1,000 bp) (for review, see [18]) and *D. melanogaster* (∼500 bp) [15]. Why should such a conserved genetic feature show these differences between taxa?

One particularly interesting evolutionary question concerns the local distribution of crossovers. Recent work in humans and mice implicates histone methyltransferase *PRDM9* as a major determinant of recombination hotspots [19]–[21], but several taxa including Drosophila lack a functional copy of this gene [22]. How are crossover locations determined in species lacking *PRDM9*? Are other histone methyltransferases playing a similar role or are crossover locations determined by other genetic features? With a detailed crossover map in *D. melanogaster*, we can begin to address this question. One motif associated with crossover locations in *D. melanogaster* is the simple repeat [CCN]_n_, which is noteworthy because the repeat [CCG]_n_ and its reverse complement [CGG]_n_ are enriched in dog recombination hotspots [7]. It is intriguing that the canine genome too lacks a functional copy of *PRDM9* [7], [22]. Further comparative work exploring crossover distribution and associated sequence motifs in humans, dogs, and Drosophila will enable great progress in uncovering the genetic determinants of crossover distribution in species lacking *PRDM9*.

These data have further implications yet, particularly for population genetic inference. Traditional population genetic models, such as those aimed at detecting selection by testing for departures from neutral expectation, rely on the fundamental assumption that recombination rate is constant within and between genomes. Violating this assumption may compromise evolutionary inferences. Previous work suggests that positive selection can lead to false inferences of recombination hotspots [23], [24], and it therefore seems reasonable to hypothesize that recombination rate heterogeneity could generate false signatures of positive selection. This hypothesis has not been tested to date, and data presented in this study informs parameter space such that we can investigate this question. Should this assumption adversely affect population genetic inference, these data will be instrumental for developing new models that accommodate recombination rate variation. Such new models have significant potential to enable robust population genetic inference of demography and adaptation.
